# The effect of calcium silicate and ground magnesium limestone (GML) on the chemical characteristics of acid sulfate soil

**DOI:** 10.1371/journal.pone.0290703

**Published:** 2023-09-15

**Authors:** Elisa Azura Azman, Roslan Ismail, Seishi Ninomiya, Shamshuddin Jusop, Uraiwan Tongkaemkaew

**Affiliations:** 1 Faculty of Agriculture, Department of Crop Science, Universiti Putra Malaysia, Serdang, Malaysia; 2 Faculty of Agriculture, Department of Land Management, Universiti Putra Malaysia, Serdang, Malaysia; 3 Department of Global Agriculture, Graduate School of Agricultural and Life Sciences, The University of Tokyo, Tokyo, Japan; 4 Faculty of Technology and Community Development, Department of Plant Science, Thaksin University, Phatthalung, Thailand; Central University of Haryana School of Life Sciences, INDIA

## Abstract

Acid sulfate soil characterized by pyrite (FeS_2_) which produces high acidity (soil pH < 3.5) and release high amount of Al^3+^ and Fe^2+^. Application of 4 t ha^-1^ Ground Magnesium Limestone (GML), is a common rate used for acid sulfate soil by the rice farmers in Malaysia. Therefore, this study was conducted to evaluate the integral effect of ground magnesium limestone (GML) and calcium silicate and to determine the optimal combination on acid sulfate soils in Malaysia. The acid sulfate soils were incubated under the submerged condition for 120 days with GML (0, 2, 4, 6 t ha^-1^) in combination with calcium silicate (0, 1, 2, 3 t ha^-1^) arranged in a Completely Randomized Design (CRD). The soil was sampled after 30, 60, 90 and 120 days of incubation and analyzed for soil pH, exchangeable Al, Ca, Mg, K and available Si. A total of 2 out of 16 combinations met the desired soil requirement for rice cultivation. The desired chemical soil characteristics for rice cultivation are soil pH > 4, exchangeable Al < 2 cmol_c_ Kg^-1^, exchangeable Ca > 2 cmol_c_ kg^-1^, exchangeable Mg > 1 cmol_c_ kg^-1^ and Si content > 43 mg kg^-1^. The combinations are i) 2 t ha^-1^ calcium silicate + 2 t ha^-1^ GML, and ii) 3 t ha^-1^ calcium silicate + 2 t ha^-1^ GML, respectively. These combination rates met the desired requirement of soil chemical characteristics for rice cultivation. Soil acidity was reduced by a gradual release of Ca^2+^ and SiO_3_^2-^ from calcium silicate continuously filling the exchange sites and reducing the potential of extra (free) H^+^ availability in the soil system. Combination of calcium silicate and GML, shows the ameliorative effect with; i) release of Ca, ii) binding of Al^3+^ making it inert Al-hydroxides and, iii) bind H^+^ to produce water molecules.

## Introduction

Acid sulfate soils are widespread in Malaysia, occurring almost exclusively along its coastal plain [1–3]. In these areas, the alluvial sediments are intermittently inundated by seawater during low and high tides. The major agronomic problems common to acid sulfate soils are toxicity due to the presence of Aluminum (Al), decrease of Phosphorus (P) availability, nutrient deficiencies, and Iron (Fe) (II) toxicity [4,5]. Thus, under normal circumstances, acid sulfate soils are not suitable for crop production, unless some amelioration practices are made. Liming the soil with Ground Magnesium Limestone (GML) is commonly used by the farmers in Malaysia. According to the [6], the application of 1.5–5 t ha^-1^ were based on soil acidity. Higher acidity soil requires more GML to neutralise the soil acidity and vice versa. The price of GML keeps increasing. For example, the price in 2010 and 2016 was USD 50 t ha^-1^ and USD 122 t ha^-1^, respectively. [7] stated that lime is not a very soluble material, and its dissociated components showed limited mobility. Considering GML’s possible drawbacks (cost and mobility limitation), we also expect that the combination of GML with calcium silicate may improve the soil’s chemical characteristics and thus improve their ameliorative effects.

Calcium silicate solubility is 6.78 times more soluble than lime [8]. [9] also reported that calcium silicate alleviates Al toxicity on acid sulfate soils of rice-cropped soil. Moreover, compared to lime alone, the advantages of silicate are higher reaction rate and mobility down to deeper soil layers.

Besides that, silicate in the form of silicon can strengthen crops against biotic and abiotic stresses on crops [10–15].

Several authors have postulated their mechanism [8,16], as shown below:

$$CaSiO_{3}\to Ca^{2+}+SiO_{3}{}^{2‐}$$ (1)

$$SiO_{3}{}^{2‐}+H_{2}O_{\left(soil\right)}\to HSiO_{3}{}^{‐}+OH^{‐}$$ (2)

$$HSiO_{3}{}^{‐}+H_{2}O_{\left(soil\right)}\to H_{2}SiO_{3}+OH^{‐}$$ (3)

$$H_{2}SiO_{3}+H_{2}O_{\left(soil\right)}\to H_{4}SiO_{4}$$ (4)

One way to reduce the acidity is by reducing the capability of H^+^ to fill in the soil exchange sites. With the addition of Ca source (calcium silicate) as soil amendments, the competition for exchange sites increase between Ca^2+^ and H^+^, and often the exchange sites are occupied by Ca^2+^. Meanwhile, the H^+^ in the soil system can be bound by SiO_3_^2-^ and becomes HSiO_3_^-^ (hydrogen silicate ion). A gradual release of Ca^2+^ and SiO_3_^2-^ from calcium silicate will continuously fill the exchange sites and reduce the potential of H^+^ availability in the soil system. With that, soil acidity can be reduced [16].

The equations release silicate (SiO_3_^2-^) ions and bind with the extra hydrogen (H^+^) ion. Further reaction progress, as shown in the equation, leads to the formation of monosilicic acid (H_4_SiO_4_), which dissociates hydroxyl ions (OH^-^). These hydroxyl ions can bind with Ca^2+^, and with continuous reaction, they will settle as Ca (OH)_2_ in the soil system. When necessary, they can dissociate and supply Ca^2+^ to the soil, and this will give a continual liming effect to the acid sulfate soils, plus calcium is a macronutrient for the plant [16].

Moreover, these free hydroxyl ions may also bind Al^3+^ to form inert Al-hydroxides (neutralise Al^3+^) and bind with H^+^ ions in the soil system and produce water molecules. Thus, with inert Al-hydroxides and minimal/less H^+^ adsorbed to the exchangeable cations capacity, the soil pH increases [17]; soil acidity decreases. Those means that the combined effects of GML and calcium silicate may have additional benefits in alleviating soil acidity and improving crop resistance.

We examined the effect of the combination of GML and calcium silicate on the improvement of acid sulfate soils and showed that the proper level combination of both showed distinct effects to achieve recommended values of selected soil chemical properties. Those values are: i) Soil pH > 4 [18], ii) Exchangeable Al < 2 cmol_c_ kg^-1^ [19], iii) Exchangeable Ca > 2 cmol_c_ kg^-1^ [20], iv) Exchangeable Mg > 1 cmol_c_ kg^-1^ [21] and v) Si content > 43 mg kg^-1^ [22].

This paper aimed; i) to evaluate the efficiency of calcium silicate with and/or without GML application on the selected soil chemical characteristics and ii) to find the optimal recommendation rate considering the positive effect of soil chemical characteristics and the costs incurred.

## Materials and methods

### Soil used in the study

Acid sulfate soils were used in this study classified as Typic Sulfaquepts, was collected from Merbok, Kedah, Peninsular Malaysia (5.7185 N, 100.3812 E). The soil sampling site was a rice-cropped area, and the sampling was performed one month before rice cultivation (dry condition). A composite soil sample of approximately 100 kg was taken from topsoil (0–15 cm) depth using an auger for submergence experiment. The sample was taken within a 0.5 ha region of the rice-cropped area. Only topsoil was collected and used for submergence study because the rice cultivation and dense root development was occurred at topsoil (0–15 cm). On the other hand, the soil was taken with a soil auger at five different depths for soil characterization purpose (Table 1). The samples were placed in plastic bags and transported back immediately to the Laboratory, Universiti Putra Malaysia for soil chemical characteristic analyses.

**Table 1:** Initial soil chemical characteristics of soils from Merbuk (Kedah).

Depth	Soil	Exchangeable cation					CEC	Extractable					Total			Al saturation
(cm)	soil: water	K	Ca	Mg	Na	Al		Fe	Cu	Zn	Mn	Si	C	N	S	%
	1:2.5	-------------------cmol_c_ kg^-1^-------------------						----------------mg kg^-1^----------------					---------%---------			
0–15	2.89	0.44	0.85	2.01	1.89	5.18	16.02	624.80	1.00	3.75	2.90	25.80	2.34	0.14	0.10	49.95
15–30	2.93	0.44	0.80	1.92	1.97	5.26	15.55	500.50	0.95	3.50	2.80	24.40	2.24	0.11	0.10	50.62
30–45	2.82	0.48	0.89	2.21	2.36	5.20	14.59	396.80	0.80	3.35	2.90	21.50	2.41	0.10	0.16	46.67
45–60	2.22	0.35	0.94	2.61	2.40	6.18	16.54	435.10	1.00	3.85	3.35	24.40	3.18	0.10	0.60	49.51
60–75	2.32	0.63	2.74	9.71	4.67	6.66	17.18	584.40	1.10	6.95	6.05	38.40	3.49	0.11	1.54	27.28

### Soil treatments and experimental design

The submergence experiment was conducted at Ladang 2, Universiti Putra Malaysia under rain shelter condition. Two types of soil amendments were used; i) ground magnesium limestone (GML) (0, 2, 4 and 6 t ha^-1^) and ii) calcium silicate (0, 1, 2 and 3 t ha^-1^) were arranged in a completely randomised design (CRD) with three replications. The total experimental unit was 192. The soil was incubated with soil amendments for 30, 60, 90 and 120 days. The GML used in this experiment was obtained from Britestone Sdn. Bhd., Malaysia. It is made of Dolomitic Limestone milled to a very fine powder with the criteria of 100% passing thru a 20-mesh screen, 70% passing thru a 100-mesh screen and more than 40% passing thru a 200-mesh screen. The chemical content of GML were calcium oxide (CaO) = 31–38%, magnesium oxide (MgO) = 15–18%, silicate oxide (SiO_2_) < 0.2% and iron oxide (Fe_2_O_3_) < 0.1%. The calcium silicate (CaSiO_3_) used in this experiment was obtained from Kaolin (Malaysia) Sdn. Bhd., Malaysia. This calcium silicate (as CaO) = 40–50%, Al_2_O_3_ = below 1.5%, MgO = below 3%, iron (as Fe_2_O_3_) = below 1% and pH = 8.54.

Five hundred grams of air-dried acid sulfate soils passed through 2 mm sieve was placed in a plastic pot. The soil samples were mixed with soil amendments (calcium silicate and ground magnesium limestone) and inundated with water. The water level was maintained at 5 cm from the soil surface throughout the experiment. The pH (pH meter PHM 93 Radiometer) of the water used was 7.37. The composition of water in relation to phosphorus (P), potassium (K), aluminium (Al), calcium (Ca), iron (Fe), magnesium (Mg), and silicon (Si) was 0.74, 10.62, 0.14, 19.78, 0.03, 1.00 and 5.18 mg L^-1^, respectively [9].

### Soil and water analysis

Soil and water sampling were carried out four times during the experiment at 30 days (30D), 60 days (60D), 90 days (90D) and 120 days (120D) correspond to typical rice growth stages; vegetative, reproductive, flowering and maturity stages, respectively. The collected soil samples were air-dried, ground and passed through a 10-mesh sieve (2 mm) for soil analyses. The following soil analyses were carried out to the collected samples: (i) Soil pH was determined in 1: 2.5 (soil to water ratio) using pH meter (PHM 93 Radiometer) [23], (ii) determination of exchangeable Al was determined by extracting 5 g soil with 50 mL of 1 M potassium chloride (KCL). The mixture was shaken for 30 min and analysed by ICP-OES (Optima 8300 ICP-OES, Perkin Elmer, Waltham, MA, USA) [23], (iii) extractable Fe, Cu, Zn and Mn were extracted using Double Dilute Acid method; with 0.05 M HCl in 0.0125 M H_2_SO_4_ in 1:5 ratios. Five (5) g of air-dried soil was mixed with 25 mL of extracting agent and shaken using electric shaker for 15 minutes at 180 rpm. The supernatant was then filtered using filter paper Whatman No. 42 and determined using Atomic Absorption Spectrometry (AAS Perkin Elmer, model 1100B) [24]; (iv) exchangeable K, Ca, Mg, Na and Fe were extracted using 1 N NH_4_Cl [18]. Briefly, 2 g of air-dried soil was put in a 50 mL centrifuge tubes and added 20 mL 1 M NH_4_Cl. After intermittent shaking for 2 hours, the tubes were centrifuged at 2500 rpm for 15 minutes. The supernatant was transferred and filtered using filter paper Whatman No. 42. The exchangeable K, Ca, Mg, Na and Fe in the extract were determined by ICP-OES (Optima 8300 ICP-OES, Perkin Elmer, Waltham, MA, USA); (v) Cation exchange capacity (CEC) of the soil was determined using 1M NH_4_OA_c_ at pH7 [25]; (vi) meanwhile, Si was extracted using 0.01 M CaCl_2_ proposed by [22]. Two (2) g of air-dried soil was shaken for 16 hours with 20 mL extractant in a 50 mL Nalgene tube using an end-over-end shaker. After centrifuging at 2000 rpm for 10 minutes, the supernatant was analysed for Si using ICP-OES (Optima 8300 ICP-OES, Perkin Elmer, Waltham, MA, USA) and (vii) Total C, N and S were determined using CNS Analyzer (Leco RC-412C Leco Corporation, St. Joseph MI) [24].

Collected water samples were filtered using filter paper Whatman No.42. Water pH was determined using a pH meter (PHM 93 Radiometer). The concentration of Al was determined using ICP-OES (Optima 8300 ICP-OES, Perkin Elmer, Waltham, MA, USA).

### Statistical analysis

Data from the experiment were analysed statistically using analysis of variance (ANOVA) and response surface curve, correlation, polynomial regression and multiple comparisons (Tukey’s test) were employed using a statistical package, Statistical Analysis System (SAS) v 9.1.

## Results

### Soil pH

**Fig 1:**
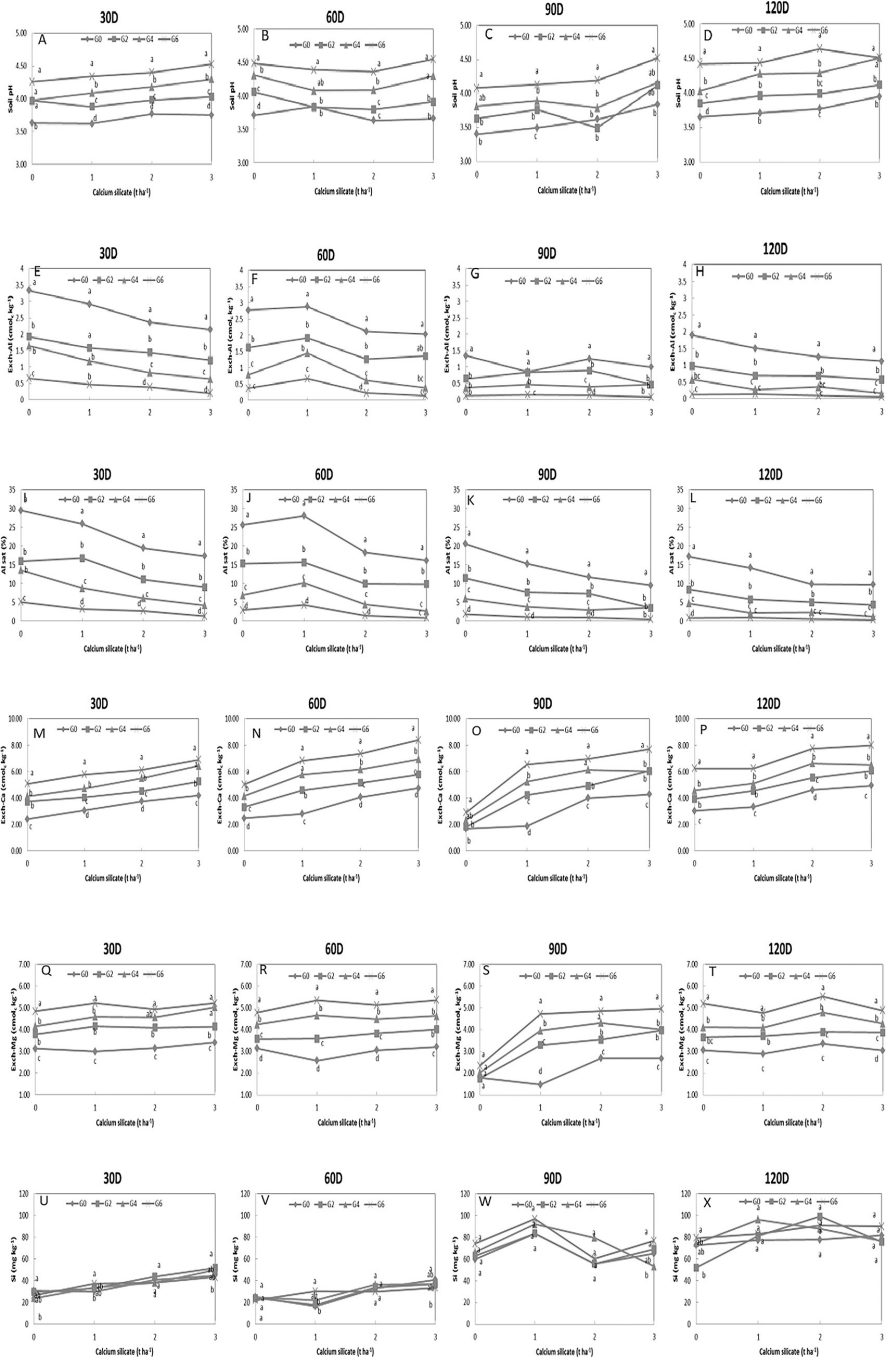
Selected soil chemical properties under the different rate of calcium silicate incorporated with GML. Means marked with the same letter for each calcium silicate treatments are not significantly different at *p* < 0.05 (Tukey`s Test). (A-D) soil pH. (E-H) exchangeable Al. (I-L) Al saturation. (M-P) exchangeable Ca. (Q-T) exchangeable Mg. (U-X) Si content.

Fig 1 shows the selected soil chemical properties under different rate of calcium silicate incorporate with GML. Initial soil pH was pH 2.89. Soil pH increased with days of incubation of 30D, 60D, 90D and 120D for Fig 1A, 1B, 1C and 1D, respectively. The soil pH ranging from pH 3.40–4.64. It was observed that the soil pH gradually increased with the increment in the rate of GML incorporated with calcium silicate. At 30D (Fig 1A), the soil treated with 2, 4 and 6 t ha^-1^ of GML under each of 0, 1, 2 and 3 t ha^-1^ of calcium silicate significantly increased the soil pH compared to the soil pH without GML. At 60D (Fig 1B), the soil that received 2 and 6 t ha^-1^ of GML significantly increased the soil pH under 0 and 1 t ha^-1^ of calcium silicate, respectively compared to the soil pH without GML. The soil pH at 90D (Fig 1C) slightly decreased under all the treated soil compared to the soil pH in 30D (Fig 1A) and 60D (Fig 1B). In comparison to the soil without GML, 6 t ha^-1^ GML significantly increased the soil pH with the combination of 0, 2 and 3 t ha^-1^ of calcium silicate. Further increases in soil pH were observed at 120D (Fig 1D). The soil with 2 t ha^-1^ GML significantly increased the soil pH compared to the soil without GML under 0 t ha^-1^ calcium silicate.

### Exchangeable Al and Al saturation

Initially, the exchangeable Al and Al saturation were 5.18 cmol_c_ kg^-1^ and 49.95%, respectively. The exchangeable Al at 30D (Fig 1E), 60D (Fig 1F), 90D (Fig 1G), 120D (Fig 1H) and Al saturation at 30D (Fig 1I), 60D (Fig 1J), 90D (Fig 1K), 120D (Fig 1L) (Fig 1I–1L) were reduced after the addition of the soil amendments corresponding with the incubation period. Under 0, 2 and 3 t ha^-1^ of calcium silicate, the soil treated with 2, 4, and 6 t ha^-1^ of GML significantly decreased the exchangeable Al compared to the soil without GML at 30D (Fig 1E). On the other hand, the soil under 1 t ha^-1^ calcium silicate significantly decreased the exchangeable Al in-combination with 4 and 6 t ha^-1^ GML compared to 0 and 2 t ha^-1^ GML. In comparison to the soil without GML, the soil with 2, 4, and 6 t ha^-1^ were significantly decreased the exchangeable Al under 0, 1 and 2 t ha^-1^ of calcium silicate at 60D (Fig 1F). The exchangeable Al values in the soil without GML were significantly higher compared to the other GML treatments at 90D (Fig 1G). Meanwhile, at 120D (Fig 1H), the exchangeable Al significantly reduced for 2, 4 and 6 t ha^-1^ of GML compared to the soil without GML under each of calcium silicate treatments (0, 1, 2 and 3 t ha^-1^). When compared to soil without GML, the soil treated with 2, 4 and 6 t ha^-1^ of GML significantly reduced the Al saturation with the combination of each calcium silicate application for the entire incubation period. The Al saturation was 49.95% before the incubation and below 35% at 30D (Fig 1H). The Al saturation in soil without GML were significantly higher compared to other GML treatments at 30D (Fig 1I), 60D (Fig 1J), 90D (Fig 1K) and 120D (Fig 1L). The most significant differences in the Al saturation occurred during the first 30 days as shown in Fig 1I, and the rate of decrease was the higher with, the higher the GML content. The Al saturation was observed reaching to 0% at 120 D as shown in Fig 1L.

### Exchangeable Ca and Mg

The soil treated with calcium silicate and GML recorded an increase of exchangeable Ca and Mg in the soil. Fig 1M, 1N, 1O and 1P show the increase of the exchangeable Ca by GML and calcium silicate addition for the entire incubation period of 30D, 60D, 90D and 120D, respectively. Compared to the soils without GML, significant increases in exchangeable Ca were observed under the GML treatments with the different rate (0, 1, 2, 3 t ha^-1^) of calcium silicate addition at 30D (Fig 1M), 60D (Fig 1N) and 120D (Fig 1P), respectively. On the other hand, Fig 1Q, 1R, 1S and 1T show the exchangeable Mg for 30D, 60D, 90D and 120D of incubation, respectively. Exchangeable Mg significantly increased with the soil treated with GML (2, 4, 6 t ha^-1^) under the different rates of calcium silicate compared to GML at 30D (Fig 1Q) and 60D (Fig 1R).

### Si content

The initial Si value was 25.8 mg kg^-1^ in the soil. Table 2 shows the Si value ranges of each incubation day summarised based on Fig 1U, 1V, 1W and 1X at 30D, 60D, 90D and 120D days of incubation, respectively. The increment of the Si values was marked with the increase in the days of incubation. A sigmoid (s-curve) increment trend was noted. In both reproductive and flowering stages, the Si content ranges were higher than 43 mg kg^-1^ under any combinations of the soil amendments while a combination level (3 t ha^-1^ calcium silicate) achieved the value in the vegetative stage. The result indicates that the soil amendment (calcium silicate) has the potential to release sufficient Si to the soil for plant uptake at least at the 60^th^ day. The released Si is expecting to be in the available form, and this form complements well with the crop requirements.

**Table 2:** Summary of the Si values (ranges and means) of each incubation days. Means marked with the same letter are not significantly different at *p* < 0.05 (Tukey’s Test).

Stage	Days of incubation (D)	Si (mg kg^-1^)	Means value
1 month after soil amendments application	30D	23.80–50.12	37.19^c^
Vegetative stage	60D	16.46–40.81	29.00^d^
Reproductive stage	90D	55.35–96.86	70.78^b^
Flowering stage	120D	52.06–96.33	81.01^a^

## Discussion

### Soil acidity reduction with time

**Fig 2:**
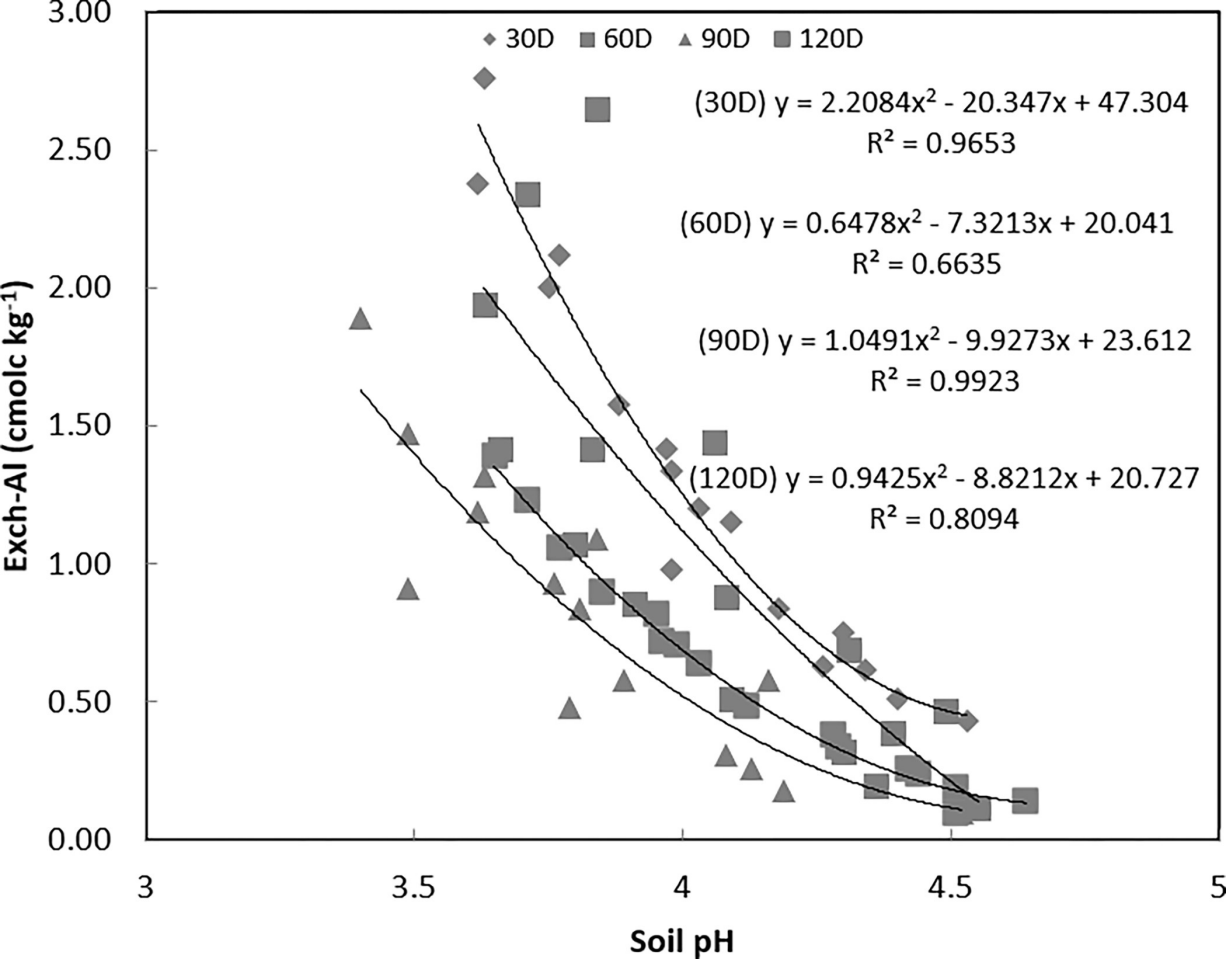
Relationship between exchangeable Al against soil pH. Polynomial regressions were conducted for the curves.

Fig 2 shows the relationship between exchangeable Al and soil pH for the entire incubation period. Exchangeable Al negatively correlated with the soil pH. Exchangeable Al decrease as the soil pH increased. It shows that the lines at 60D, 90D and 120D were shifted to the left. Fig 2 shows the line shift to the left indicates that Al toxicity decreased as the incubation period increased. The line at 90D was below 120D, and this is believed due to the release of protons as pyrite in the soil was oxidised during the incubation period. The oxidation of pyrite, which produces acidity, may have taken place according to the following reactions outlined by [26]:

$$2\;FeS_{2(s)}+7\;O_{2\;(aq)}+2\;H_{2}O\to Fe^{2+}{}_{\left(aq\right)}+4\;SO_{4}{}^{2‐}+4\;H^{+}{}_{\left(aq\right)}$$ (5)

Further oxidation of Fe^2+^ to Fe^3+^ oxide could also promote acidity:

$$2\;Fe^{2+}{}_{\left(aq\right)}+½\;O_{2(aq,g)}+H_{2}O\to Fe_{2}O_{3(s)}+4H^{+}{}_{\left(aq\right)}$$ (6)

The results from the current study are consistent with those from other studies of acid sulfate soils [1,2,27]. It is shown in Eqs (1) and (2) that one mole of pyrite produces four moles of sulfuric acid. This free acidity is partly responsible for the dissolution of clay minerals, resulting in the release of metals like Al into the soils, shown by Fig 1H. Furthermore [28], reported that soil pH in the Cg horizon (subsoil) was lowered by 1 unit after 12 weeks of incubation.

**Fig 3:**
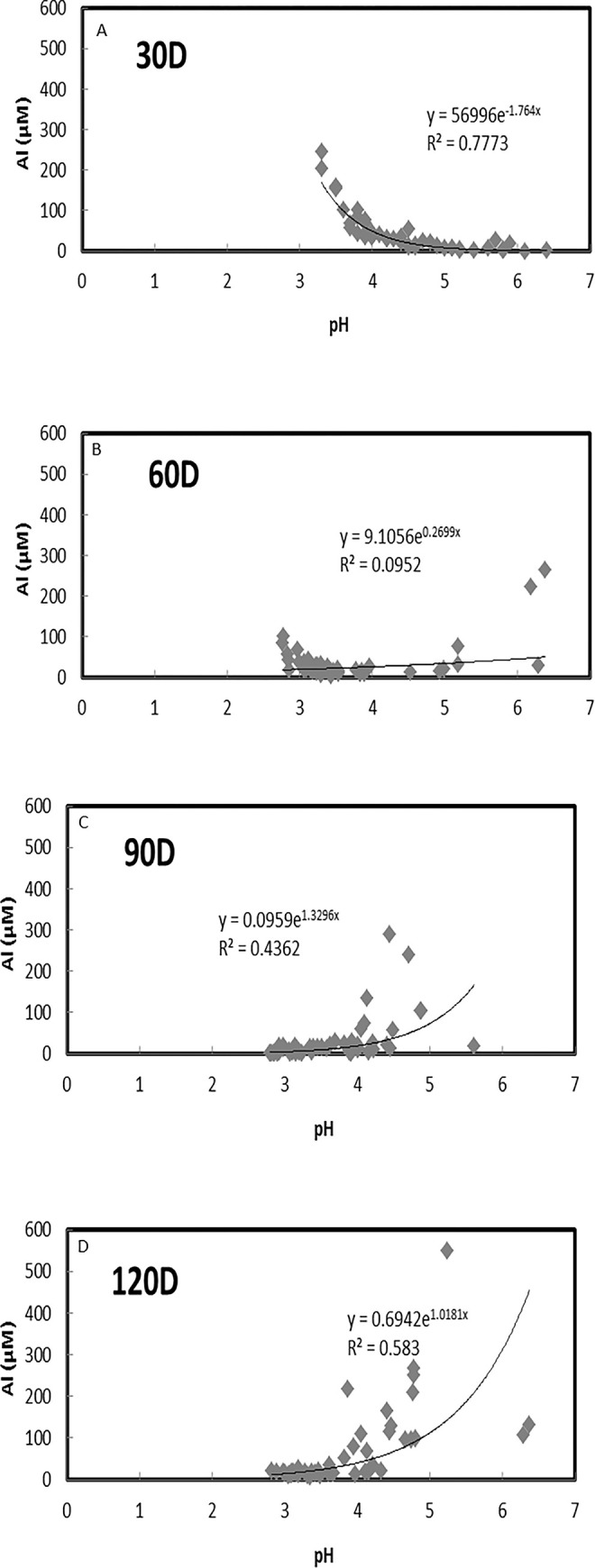
Relationship between Al_water_ concentration and pH of water solution. (A) after 30 days. (B) after 60 days. (C) after 90 days. (D) after120 days.

The soil pH (Fig 2) did not exceed pH 5 compared to the water solution pH, as shown in Fig 3. Fig 3 shows the relationship between Al_water_ concentration and pH of water solution. Fig 3A, 3B, 3C and 3D show the relationship between Al_water_ and pH at 30D, 60D, 90D and 120D days of incubation, respectively. At 30D (Fig 3A), Al_water_ concentration decreased with the increment in the water solution pH. The Al_water_ concentration was observed to gradually increase at 60D (Fig 3B), 90D (Fig 3C) and 120D (Fig 3D). Their gradual improvement will support positive crop growth inline with vegetative (30D), reproductive (60D), flowering (90D) and maturity (120D) paddy stages.

### GML and calcium silicate combined ameliorative effects on the selected soil characteristics

Firstly, the chemical mechanism of soil amendments used in this study was discussed regarding their effects in alleviating soil acidity and improving soil fertility. After that, the soil chemical characteristics at 30^th^ days of soil incubation were presented. Finally, the most optimal combination of the application levels of both calcium silicate and GML were selected as a recommendation rate to the farmers at the respective area in Malaysia. The recommendation rate was evaluated based on the following factors.

- The positive effect of chemical soil characteristics at the 30^th^ day of incubation.
  There are two reasons to focus on the data from the 30 days of the incubation. First, the acidity gradually decreases in soil (Fig 2) and water (Fig 3). This will support positive crop growth inline with vegetative, reproductive and flowering of rice growth phases. Second, it is more time-suitable for them otherwise they need to wait too long before they start planting and;
- Cost incurs for soil amendments application.
  Because farmers are currently using GML at the rate of 4 t ha^-1,^ which costs approximately USD 668 (cost of GML only), we seek for the optimal combinations of calcium silicate and GML to reduce the cost.

Calcium silicate was used to replace calcium silicate slag due to regulations in Malaysia, Environmental Quality Act 1974, that prohibits direct use of solid waste onto the soil for crop production and other means.

It is possible to postulate five different mechanisms of Al toxicity reduction by Si-rich compounds. Firstly, monosilicic acids can increase soil pH [17]. Secondly, monosilicic acids can be adsorbed on aluminium hydroxides, impairing their mobility [29]. Thirdly, soluble monosilicic acid can form slightly soluble substances with ions of Al [30]. Another possibility for Al toxicity reduction by Si-rich compounds can be strong adsorption of mobile Al on silica surfaces [31]. Lastly, mobile silicon compounds can increase plant tolerance to Al [32]. All of these mechanisms may occur simultaneously, with certain ones prevailing under various soil conditions.

GML is well known to increase the soil pH, and release Ca and Mg into the soil system. GML ameliorative reactions are shown below:

$$(Ca,Mg){\left(CO_{3}\right)}_{2}\;\to Ca^{2+}+Mg^{2+}+2CO_{3}{}^{2‐}$$ (Eq 1)

$$CO_{3}{}^{2‐}+H_{2}O\;\to HCO_{3}{}^{‐}+OH^{‐}$$ (Eq 2)

$$Al^{3+}+3\;OH^{‐}\;\to Al\;{\left(OH\right)}_{3}$$ (Eq 3)

GML dissolves gradually into the soil, and releases Ca and Mg (Eq 1), and these macronutrients could be taken up by the growing rice plants. Subsequently, the hydrolysis of CO_3_^2-^ (Eq 2) would produce hydroxyls that neutralise Al by forming inert Al-hydroxides (Eq 3). Combination of calcium silicate and GML, both shows the significant ameliorative effect with; i) release of Ca, ii) binding of Al^3+^ making it inert Al-hydroxides and, iii) bind H^+^ to produce water molecules.

**Fig 4:**
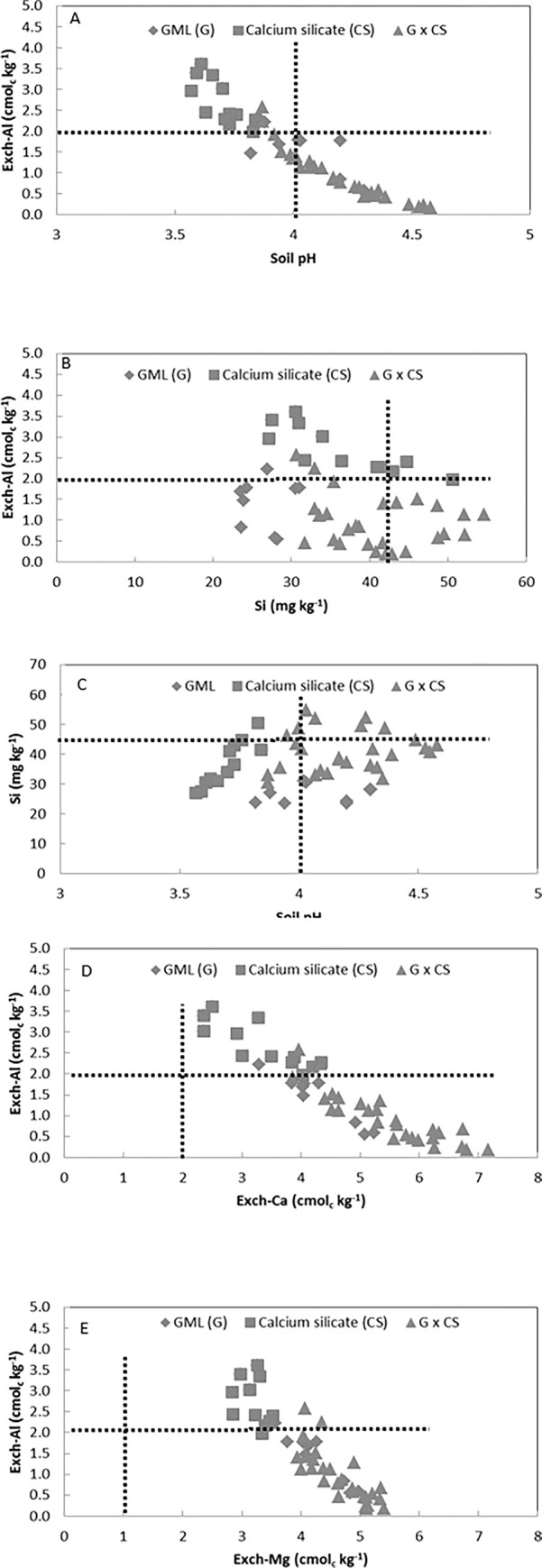
Relationship between soil chemical characteristic at the 30^th^ day of the incubation. Dotted lines indicate the critical value of soil chemical parameter. (A) exchangeable Al and soil pH. (B) exchangeable Al and Si content. (C) Si and soil pH. (D) exchangeable Al and exchangeable Ca. (E) exchangeable Al and exchangeable Mg.

Fig 4A shows that exchangeable Al decreased with increment in soil pH. The addition of calcium silicate alone could reduce the exchangeable Al below the critical value (< 2 cmol_c_ kg^-1^) and soil pH higher than 4 to avoid their inhibitory effects on rice growth. The distribution pattern shifted to the right when the soil was treated with both GML and calcium silicate, indicating the combined ameliorative effects of both soil amendments. Under most of the combinations of both amendments with different application levels, the exchangeable Al and soil pH values fall within the critical values (Al < 2 cmol_c_ kg^-1^ and soil pH > 4). Thus, the result indicates that the addition of both soil amendments improved the acid sulfate soils compared to single soil amendment application.

Fig 4B shows silicon content was negatively correlated with exchangeable Al under application of calcium silicate only, keeping the Al level higher than the critical value of 2 cmol_c_ kg^-1^. The Si content was lower than sufficient level (> 43 mg kg^-1^) for crop growth only with GML though the exchangeable Al is lower than the critical value. Direct correlation of Si in soil solution with Al phytotoxicity in soil solution was recorded by [33]; Si content increase while Al decrease. These results suggest that the interaction between aluminium and silicon occur in solution, probably by the formation of a complex between aluminium and silicon that is not toxic to plants. Studies conducted by [9] and [34] showed that Si could effectively reduce Al toxicity. Under a proper combination of application levels of the both of the amendments, exchangeable Al were lower than 2 cmol_c_ kg^-1^, while Si contents > 43 mg kg^-1^compared to the application of GML or calcium silicate only.

Fig 4C shows soil pH positively correlates with silicon content under the calcium silicate application alone. The application of one type of soil amendments alone, either calcium silicate or GML, was unable to increase the soil pH above 4 and the silicon content above 43 mg kg^-1^, respectively. Statistically, no significant relationship between soil pH and silicon content was observed for soil treated with the only GML and with the combination of both soil amendments. However, the distribution pattern shifted to the right in the soil with GML and the combination of calcium silicate and GML. Some of the combined applications were able to fulfil above the recommended levels.

Fig 4D shows that exchangeable Ca negatively correlates with exchangeable Al. As the exchangeable Ca increased, the exchangeable Al decreased. Single or combined application of soil amendments made the exchangeable Ca reach the requirement of 2 cmol_c_ kg^-1^. However, the application of calcium silicate alone did not reduce the exchangeable Al below the critical level of 2 cmol_c_ kg^-1^ while the exchangeable Al was reduced below the critical level for application of GML alone and the combination.

Fig 4E shows that exchangeable Mg increased as the exchangeable Al decreased. The addition of calcium silicate alone did not decrease the exchangeable Al below the critical level of 2 cmol_c_ kg^-1^. On the other hand, the addition of GML alone or the combination of both soil amendments decreased the exchangeable Al below the critical level of 2 cmol_c_ kg^-1^. The application of the soil amendments alone or with the combination increased in exchangeable Mg above the required level of 1 cmol_c_ kg^-1^.

### Effective combination rate of calcium silicate and GML as soil amendments

The combination rate in shaded area achieved the recommended level for soil pH (Table 3A), exchangeable Al (Table 3B), exchangeable Ca (Table 3C), exchangeable Mg (Table 3D) and Si content (Table 3E) of > 4 [18], < 2 cmol_c_ kg^-1^ [19], > 2 cmol_c_ kg^-1^ [20], > 1 cmol_c_ kg^-1^ [21] and > 43 mg kg^-1^ [22], respectively.

**Table 3:** Effect of calcium silicate in-combination with GML on (A) soil pH, (B) exchangeable Al, (C) exchangeable Ca, (D) exchangeable Mg and (E) Si content at 30D. Means marked with the same letter for each calcium silicate treatments are not significantly different at *p* < 0.05 (Tukey`s Test) (The combination rate in shaded area achieved the recommended level of each chemical soil characteristic).

**A**				
	**Calcium silicate (t ha** ^**-1**^ **)**			
**GML (t ha** ^**-1**^ **)**	**0**	**1**	**2**	**3**
**0**	**3.63** ^**b**^	**3.62** ^**d**^	**3.77** ^**d**^	**3.75** ^**d**^
**2**	**3.97** ^**a**^	**3.88** ^**c**^	**3.98** ^**c**^	**4.03** ^**c**^
**4**	**3.98** ^**a**^	**4.09** ^**b**^	**4.18** ^**b**^	**4.30** ^**b**^
**6**	**4.26** ^**a**^	**4.34** ^**a**^	**4.40** ^**a**^	**4.53** ^**a**^
**B**				
	**Calcium silicate (t ha** ^**-1**^ **)**			
**GML (t ha** ^**-1**^ **)**	**0**	**1**	**2**	**3**
**0**	**3.34** ^**a**^	**2.91** ^**a**^	**2.36** ^**a**^	**2.14** ^**a**^
**2**	**1.92** ^**b**^	**1.58** ^**a**^	**1.44** ^**b**^	**1.20** ^**b**^
**4**	**1.64** ^**b**^	**1.17** ^**b**^	**0.82** ^**c**^	**0.63** ^**c**^
**6**	**0.65** ^**c**^	**0.46** ^**b**^	**0.38** ^**d**^	**0.20** ^**d**^
**C**				
	**Calcium silicate (t ha** ^**-1**^ **)**			
**GML (t ha** ^**-1**^ **)**	**0**	**1**	**2**	**3**
**0**	**2.41** ^**c**^	**3.06** ^**d**^	**3.75** ^**d**^	**4.19** ^**c**^
**2**	**3.73** ^**b**^	**4.05** ^**c**^	**4.53** ^**c**^	**5.26** ^**b**^
**4**	**4.13** ^**b**^	**4.72** ^**b**^	**5.51** ^**b**^	**6.44** ^**a**^
**6**	**5.08** ^**a**^	**5.78** ^**a**^	**6.12** ^**a**^	**6.90** ^**a**^
**D**				
	**Calcium silicate (t ha** ^**-1**^ **)**			
**GML (t ha** ^**-1**^ **)**	**0**	**1**	**2**	**3**
**0**	**3.13** ^**c**^	**2.99** ^**c**^	**3.15** ^**c**^	**3.41** ^**c**^
**2**	**3.79** ^**b**^	**4.15** ^**b**^	**4.09** ^**b**^	**4.13** ^**b**^
**4**	**4.16** ^**b**^	**4.59** ^**b**^	**4.57** ^**ab**^	**5.03** ^**a**^
**6**	**4.84** ^**a**^	**5.22** ^**a**^	**4.94** ^**a**^	**5.21** ^**a**^
**E**				
	**Calcium silicate (t ha** ^**-1**^ **)**			
**GML (t ha** ^**-1**^ **)**	**0**	**1**	**2**	**3**
**0**	**30.65** ^**a**^	**29.90** ^**b**^	**40.84** ^**a**^	**44.82** ^**ab**^
**2**	**29.48** ^**ab**^	**33.02** ^**ab**^	**43.80** ^**a**^	**51.79** ^**a**^
**4**	**23.80** ^**b**^	**33.73** ^**ab**^	**38.09** ^**a**^	**50.12** ^**ab**^
**6**	**26.50** ^**ab**^	**37.13** ^**a**^	**38.08** ^**a**^	**43.21** ^**b**^

Finally, Table 4 shows the soil chemical characteristics which meet the recommended level for each combination of calcium silicate and GML. It shows that a combination of 3 t ha^-1^ calcium silicate with 2, 4 or 6 t ha^-1^ GML achieved the recommended levels for all the five soil chemical characteristics of, the soil pH, exchangeable Al, Ca, Mg and Si content (marked in light shaded area). The combination of 2 t ha^-1^ calcium silicate with 2 t ha^-1^ of GML achieved the recommended levels of 4 soil chemical characteristics out of 5 (marked in dark shaded area), the exchangeable Al, Ca, Mg and Si content. Though the recommended soil pH of 4 was not achieved in this combination, the value, pH 3.98 was very close to 4. The combined rate of GML, 2 t ha^-1^ calcium silicate with 4 t ha^-1^ GML, 2 t ha^-1^ calcium silicate with 6 t ha^-1^ GML, 1 t ha^-1^ calcium silicate with 4 t ha^-1^ GML and 2 t ha^-1^ calcium silicate with 6 t ha^-1^ GML achieved the recommended levels of 4 soil chemical characteristics out of 5 (marked in dark shaded area), the soil pH, exchangeable Al, Ca and Mg. In those combinations, Si was below the recommended value of 43 mg kg^-1^. The remaining combination rate of calcium silicate and GML achieved 3 or less of the recommended level for soil chemical characteristics.

**Table 4:** Combination of calcium silicate with GML achieves the recommended value of soil chemical characteristics. (Combination rate achieved 4 out of 5 recommended level marked with dark shaded area while the combination rate achieved five recommended level marked with light shaded area).

	Calcium silicate (t ha^-1^)			
GML (t ha^-1^)	0	1	2	3
**0**	**Ca** **Mg**	**Ca** **Mg**	**Ca** **Mg** **Si**	**Ca** **Mg** **Si**
**2**	**Al** **Ca** **Mg**	**Al** **Ca** **Mg**	**Al** **Ca** **Mg** **Si**	**pH** **Al** **Ca** **Mg** **Si**
**4**	**Al** **Ca** **Mg**	**pH** **Al** **Ca** **Mg**	**pH** **Al** **Ca** **Mg**	**pH** **Al** **Ca** **Mg** **Si**
**6**	**pH** **Al** **Ca** **Mg**	**pH** **Al** **Ca** **Mg**	**pH** **Al** **Ca** **Mg**	**pH** **Al** **Ca** **Mg** **Si**

The combination of calcium silicate and GML, which achieved 4 and 5 of recommended levels were selected. Those combination rates were further analysed for the feasibility analysis to find out the most optimal combination balancing the fertility improvement and the cost-effectiveness.

### Feasibility analysis

Table 5 shows the cost incurs for soil amendments application. The cost includes the price of soil amendments and labour cost. Currently, the price for both calcium silicate slag and GML is USD 30 t^-1^ and USD 122 t^-1^, respectively, while the labour cost incurs at USD 45 t^-1^. Currently, farmers at the respective area use 4 t of GML ha^-1^ with the cost of USD 668 (marked in light shaded area). Therefore, the total cost of less than USD 668 was considered. From Table 5, it shows that 9 out of 16 combinations achieved 4 or 5 of the recommended level for soil chemical characteristics. However the costs for the combination of 2 t ha^-1^ calcium silicate with 2 t ha^-1^ GML and 3 t ha^-1^ calcium silicate with 2 t ha^-1^ GML were less from USD 668; USD 484 and USD 559, respectively (Table 5) (marked in dark shaded area).

**Table 5:** Cost incurs for application of soil amendments (light shaded area indicate the common practice while dark shaded area indicate total cost below the common practice and achieved 4 or 5 recommended level of soil chemical characteristics.

	Calcium silicate (t ha^-1^)			
GML (t ha^-1^)	0	1	2	3
**0**	**SA: 0** **L: 0** **T:0**	**SA: 30** **L: 45** **T: 75**	**SA: 60** **L: 90** **T: 150**	**SA: 90** **L: 135** **T:225**
**2**	**SA: 244** **L: 90** **T: 334**	**SA: 274** **L: 135** **T:409**	**SA: 309** **L: 180** **T: 484**	**SA: 334** **L: 225** **T: 559**
**4**	**SA: 488** **L: 180** **T: 668**	**SA: 518** **L: 225** **T:743**	**SA: 548** **L: 270** **T: 818**	**SA: 334** **L: 225** **T: 559**
**6**	**SA: 732** **L: 270** **T: 1002**	**SA: 762** **L: 315** **T: 1077**	**SA: 792** **L: 360** **T: 1152**	**SA: 882** **L: 405** **T:1227**

**SA: Soil amendments (GML cost at USD 122 t**^**-1**^ **and calcium silicate cost at USD 30 t**^**-1**^**).**

**L: Labor cost at USD 45 t**^**-1**^.

T: Total cost of SA+L.

Out of the possible two recommendations, the combination of 3 t ha^-1^ calcium silicate with 2 t ha^-1^ GML achieved the recommended levels of all the targeted soil characteristics. The costs differences between the combination and the common practice with 4 t ha^-1^ of GML are USD 154 (USD 488—USD 334) for only the soil amendment cost and USD 109 (USD 668—USD 559) for the total cost including labor cost, meaning that this recommendation is USD 114 more beneficial per ha (16% less) for the farmers.

Another possible recommendation of 2 t ha^-1^ calcium silicate with 2 t ha^-1^ GML did not achieve the pH 4 but the recommended levels of the exchangeable Al, Ca, Mg and Si content. The pH level of the combination was, however, pH 3.98, which was very close to the recommended level of 4. In this combination, the costs differences between the combination and the common practice with 4 t ha^-1^ of GML are USD 184 (USD 488—USD 304) for only the soil amendment cost and USD 184 (USD 668—USD 484) for the total cost including labor cost, meaning that this recommendation is USD 184 more beneficial per ha (28%) for the farmers. In this study, the pH level under the standard practice with 4 t ha^-1^ of GML was also 3.98 (Table 3A) which is below the recommended level and the same as that of this recommendation. Moreover, under the condition of the common practice, Si did not achieve the recommended level of 43 mg kg^-1^ (Table 3E), indicating that the combination of 2 t ha^-1^ calcium silicate with 2 t ha^-1^ GML may improve rice growth better than the standard practice in addition to the cost reduction of USD 184. Therefore, the combination can be advantageous for the farmers despite the pH level.

Both of the recommendations are more beneficial than conventional practice. In term of the total cost, the combination of 2 t ha^-1^ calcium silicate with 2 t ha^-1^ GML is better than the combination of 3 t ha^-1^ calcium silicate with 2 t ha^-1^ GML, though the pH level is below 4 for the former case. If farmers can expect that improvement of the yield under the later combination compensates the cost difference between two recommendations, the choice can be the latter. At this moment, we do not have the information about the relationship between the combination and the yield, and we need further studies to conclude the recommendation, considering the expected yield. With that information, the combinations which achieved the recommended levels of 5 soil characteristics (3 t ha^-1^ calcium silicate with 4 t ha^-1^ GML and 3 t ha^-1^ calcium silicate with 6 t ha^-1^ GML) can be consider to produce higher yield and this may be able to compensate the cost increase.

## Conclusion

The possible recommendation rate is 2 t ha^-1^ calcium silicate with 2 t ha^-1^ GML price at USD 484 and 3 t ha^-1^ calcium silicate with 2 t ha^-1^ GML price at USD 559. That recommendation rate achieved the recommended levels of soil pH > 4, exchangeable Al < 2 cmol_c_ Kg^-1^, exchangeable Ca > 2 cmol_c_ kg^-1^, exchangeable Mg > 1 cmol_c_ kg^-1^ and Si content > 43 mg kg^-1^ with the total cost below than common practice of USD 668 in Malaysia. The future prospect or recommendation is to apply these combination on the farmers’ rice field and planting with rice to determine the rice yield performances.

## References

[1] Shamshuddin J. and Auxtero E., “Soil solution compositions and minerology of some active aci sulfate soils in Malaysia as affected by laboratory incubation with lime,” *Soil Sci*., vol. 152, no. 5, 1991, [Online]. Available: https://journals.lww.com/soilsci/Fulltext/1991/11000/SOIL_SOLUTION_COMPOSITIONS_AND_MINERALOGY_OF_SOME.8.aspx.

[2] Shamshuddin J., Jamilah I., and Ogunwale J. A., “Formation of hydroxy-sulfates from pyrite in coastal acid sulfate soil environments in malaysia,” *Commun*. *Soil Sci*. *Plant Anal*., vol. 26, no. 17–18, pp. 2769–2782, Oct. 1995, doi: 10.1080/00103629509369486

[3] Enio M. S. K., Shamshuddin J., Fauziah C. I., and Husni M. H. A., “Pyritization of the coastal sediments in the Kelantan plains in the Malay peninsula during the Holocene,” *Am*. *J*. *Agric*. *Biol*. *Sci*., vol. 6, no. 3, pp. 393–402, Aug. 2011, doi: 10.3844/ajabssp.2011.393.402

[4] Dent D., *Acid sulphate soils*: *a baseline for research and development*. Wageningen, The Netherlands: International Institute for Land Reclamation and Improvement, 1986.

[5] Elisa A. A., Shamshuddin J., and Fauziah C. I., “Root elongation, root surface area and organic acid by rice seedling under Al 3+ and/or H + stress,” *Am*. *J*. *Agric*. *Biol*. *Sci*., vol. 6, no. 3, pp. 324–331, 2011, doi: 10.3844/ajabssp.2011.324.331

[6] M. Department of Agriculture Malaysia, Ministry of Agriculture, *Manual tanaman padi (Rice planting handbooks*, *manual)*. Perpustakaan Negara Malaysia, 2006.

[7] Soratto R. P. and Crusciol C. A. C., “Dolomite and Phosphogypsum Surface Application Effects on Annual Crops Nutrition and Yield,” *Agron*. *J*., vol. 100, no. 2, pp. 261–270, Mar. 2008, doi: 10.2134/agronj2007.0120

[8] Alcarde J. and Rodella A., “Quality and legislations of fertilizer and acidity correction sourcesNo Title,” in *Topics in Soil Science*, Curi N., Marques J., Guilherme L., Lima J., Lopes A., and Alvares V., Eds. 2003, pp. 291–334.

[9] Elisa A. A., Ninomiya S., Shamshuddin J., and Roslan I., “Alleviating aluminum toxicity in an acid sulfate soil from Peninsular Malaysia by calcium silicate application,” *Solid Earth*, vol. 7, no. 2, pp. 367–374, 2016, doi: 10.5194/se-7-367-2016

[10] Peaslee D. E. and Frink C. R., “Influence of Silicie Acid on Uptake of Mn, Al, Zn, and Cu by Tomatoes (Lycopersicum esculentum) Grown on an Acid Soil,” *Soil Sci*. *Soc*. *Am*. *J*., vol. 33, no. 4, pp. 569–571, Jul. 1969, doi: 10.2136/sssaj1969.03615995003300040025x

[11] Menzies J., Bowen P., Ehret D., and Glass A. D. M., “Foliar Applications of Potassium Silicate Reduce Severity of Powdery Mildew on Cucumber, Muskmelon, and Zucchini Squash,” *J*. *Am*. *Soc*. *Hortic*. *Sci*., vol. 117, no. 6, pp. 902–905, Jan. 2019, doi: 10.21273/jashs.117.6.902

[12] Hodson M. J. and Evans D. E., “Aluminium/silicon interactions in higher plants,” *Journal of Experimental Botany*, vol. 46, no. 2. Oxford Academic, pp. 161–171, Feb. 01, 1995, doi: 10.1093/jxb/46.2.161 PMC7709911 31950161

[13] Romero-Aranda M. R., Jurado O., and Cuartero J., “Silicon alleviates the deleterious salt effect on tomato plant growth by improving plant water status,” *J*. *Plant Physiol*., vol. 163, no. 8, pp. 847–855, Jul. 2006, doi: 10.1016/j.jplph.2005.05.010 16777532

[14] Liang Y., Sun W., Zhu Y. G., and Christie P., “Mechanisms of silicon-mediated alleviation of abiotic stresses in higher plants: A review,” *Environ*. *Pollut*., vol. 147, no. 2, pp. 422–428, May 2007, doi: 10.1016/j.envpol.2006.06.008 16996179

[15] Abed-Ashtiani F., Kadir J.-B., Selamat A.-B., Husni A., Hanif B.-M., and Nasehi A., “Effect of Foliar and Root Application of Silicon Against Rice Blast Fungus in MR219 Rice Variety,” *Plant Pathol*. *J*, vol. 28, no. 2, pp. 164–171, 2012, doi: 10.5423/PPJ.OA.02.2012.0022

[16] Nolla A., Henrique Korndörfer G., Amaral Tavares Da Silva C, Roque Benetoli Da Silva T, Zucarelli V, and Anita Gonçalves Da Silva M, “Correcting soil acidity with the use of slags,” vol. 8, no. 41, pp. 5174–5180, 2013, doi: 10.5897/AJAR2013.6940

[17] Lindsay W., Ed., *Chemical equilibria in soils*. 1979.

[18] Shamshuddin J., *Acid Sulfate Soils in Malaysia*. Serdang, Selangor: Universiti Putra Malaysia Press, 2006.

[19] Hiradate S., Ma J. F., and Matsumoto H., “Strategies of Plants to Adapt to Mineral Stresses in Problem Soils,” *Advances in Agronomy*, vol. 96. Academic Press, pp. 65–132, Jan. 01, 2007, doi: 10.1016/S0065-2113(07)96004-6

[20] Palhares de Melo L. A. M., Bertioli D. J., Cajueiro E. V. M., and Bastos R. C., “Recommendation for fertilizer application for soils via qualitative reasoning,” *Agric*. *Syst*., vol. 67, no. 1, pp. 21–30, Jan. 2001, doi: 10.1016/S0308-521X(00)00044-5

[21] Dobermann A. and Fairhurst T., *Nutrient Disorders & Nutrient Management Rice Rice ecosystems Nutrient management Nutrient deficiencies Mineral toxicities Tools and information*. 2000.

[22] Narayanaswamy C. and Prakash N. B., “Calibration and categorization of plant available silicon in rice soils of South India,” *J*. *Plant Nutr*., vol. 32, no. 8, pp. 1237–1254, Aug. 2009, doi: 10.1080/01904160903005970

[23] Shamshuddin J., Panhwar Q. A., Shazana M. A. R. S., Elisa A. A., Fauziah C. I., and Naher U. A., “Improving the productivity of acid sulfate soils for rice cultivation using limestone, basalt, organic fertilizer and/or their combinations,” *Sains Malaysiana*, vol. 45, no. 3, pp. 383–392, 2016.

[24] Carter M. R. and Gregorich E. G., *Methods of Analysis Second Edition Soil Sampling and*, Second. United States of America: CRC Press, 2008.

[25] Chapman H. D., “Cation-Exchange Capacity,” in *Agronomy Journal*, vol. 9, John Wiley & Sons, Ltd, 2016, pp. 891–901.

[26] van Breemen N., “Genesis and solution chemistry of acid sulfate soils in Thailand,” Wageningen, 1976. Accessed: Sep. 23, 2020. [Online]. Available: https://library.wur.nl/WebQuery/wurpubs/70246.

[27] Shamshuddin J., Elisa Azura A., Shazana M. A. R. S., Fauziah C. I., Panhwar Q. A., and Naher U. A., *Properties and management of acid sulfate soils in Southeast Asia for sustainable cultivation of rice*, *oil palm*, *and cocoa*, vol. 124. 2014.

[28] Shamshuddin J., Muhrizal S., Fauziah I., and Van Ranst E., “A Laboratory Study of Pyrite Oxidation in Acid Sulfate Soils,” *Commun*. *Soil Sci*. *Plant Anal*., vol. 35, no. 1–2, pp. 117–129, 2004, doi: 10.1081/CSS-120027638

[29] Panov N., Goncharova N., and Rodionova L., “The role of amorphous silicic acid in solonetz soil processes,” *Vestn*. *Agr*. *Sci*., vol. 11, p. 18, 1982.

[30] Lumsdon D. G. and Farmer V. C., “Solubility characteristics of proto-imogolite sols: how silicic acid can de-toxify aluminium solutions,” *Eur*. *J*. *Soil Sci*., vol. 46, no. 2, pp. 179–186, Jun. 1995, doi: 10.1111/j.1365-2389.1995.tb01825.x

[31] Schulthess C. P. and Tokunaga S., “Metal and pH Effects on Adsorption of Poly(vinyl alcohol) by Silicon Oxide,” *Soil Sci*. *Soc*. *Am*. *J*., vol. 60, no. 1, pp. 92–98, Jan. 1996, doi: 10.2136/sssaj1996.03615995006000010016x

[32] Rahman M. T., Kawamura K., Koyama H., and Hara T., “Varietal differences in the growth of rice plants in response to aluminum and silicon,” *Soil Sci*. *Plant Nutr*., vol. 44, no. 3, pp. 423–431, 1998, doi: 10.1080/00380768.1998.10414464

[33] Cocker K. M., Evans D. E., and Hodson M. J., “The amelioration of aluminium toxicity by silicon in higher plants: Solution chemistry or an in planta mechanism?,” *Physiol*. *Plant*., vol. 104, no. 4, pp. 608–614, Dec. 1998, doi: 10.1034/j.1399-3054.1998.1040413.x

[34] Myhr K. and Erstad K., “Converter slag as a liming material on organic soils,” *Nor*. *J*. *Agric*. *Sci*., vol. 10, no. 1, pp. 83–93, 1996, Accessed: Sep. 23, 2020. [Online]. Available: https://agris.fao.org/agris-search/search.do?recordID=NO9600123.

